# Molecular serotyping of diarrheagenic *Escherichia coli* with a MeltArray assay reveals distinct correlation between serotype and pathotype

**DOI:** 10.1080/19490976.2024.2401944

**Published:** 2024-09-18

**Authors:** Chen Du, Yiqun Liao, Congcong Ding, Jiayu Huang, Shujuan Zhou, Yiyan Xu, Zhaohui Yang, Xiaolu Shi, Yinghui Li, Min Jiang, Le Zuo, Minxu Li, Shengzhe Bian, Na Xiao, Liqiang Li, Ye Xu, Qinghua Hu, Qingge Li

**Affiliations:** a Engineering Research Centre of Molecular Diagnostics of the Ministry of Education, State Key Laboratory of Cellular Stress Biology, State Key Laboratory of Molecular Vaccinology and Molecular Diagnostics, School of Life Sciences and School of Public Health, Xiamen University, Xiamen, China; b Microbiology Lab Office, Shenzhen Center for Disease Control and Prevention, Shenzhen, China; c BGI research, BGI-Shenzhen, Shenzhen, China; d Laboratory Department, Yantian District Center for Disease Control and Prevention, Shenzhen, China; e National Clinical Research Center for Infectious Diseases, The Third People’s Hospital of Shenzhen, Southern University of Science and Technology, Shenzhen, China

**Keywords:** Diarrheagenic *Escherichia coli*, *Shigella*, molecular serotyping, culture-independent serotyping, correlation between serotype and pathotype

## Abstract

Diarrheagenic *Escherichia coli* serotypes are associated with various clinical syndromes, yet the precise correlation between serotype and pathotype remains unclear. A major barrier to such studies is the reliance on antisera-based serotyping, which is culture-dependent, low-throughput, and cost-ineffective. We have established a highly multiplex PCR-based serotyping assay, termed the MeltArray *E. coli* serotyping (*EST*) assay, capable of identifying 163 O-antigen-encoding genes and 53 H-antigen-encoding genes of *E. coli*. The assay successfully identified serotypes directly from both simulated and real fecal samples, as demonstrated through spike-in validation experiments and a retrospective study. In a multi-province study involving 637 *E. coli* strains, it revealed that the five major diarrheagenic pathotypes have distinct serotype compositions. Notably, it differentiated 257 *Shigella* isolates into four major *Shigella* species, distinguishing them from enteroinvasive *E. coli* based on their distinct serotype profiles. The assay’s universality was further corroborated by *in silico* analysis of whole-genome sequences from the EnteroBase. We conclude that the MeltArray *EST* assay represents a paradigm-shifting tool for molecular serotyping of *E. coli*, with potential routine applications for comprehensive serotype analysis, disease diagnosis, and outbreak detection.

## 1. Introduction

Diarrheal illnesses and foodborne outbreaks caused by diarrheagenic *Escherichia coli* (DEC) are enduring public health threats, and the major causes of morbidity and mortality in children in developing countries in particular.^1–3^ However, not all *E. coli* strains isolated from diarrheal patients are pathogenic because commensal *E. coli* also extensively colonize the gut,^4–6^ Therefore, distinguishing pathogenic strains from nonpathogenic ones is critical for controlling DEC infections.

*E. coli* pathotypes are classified based on the presence of specific virulence genes,^7^ while *E. coli* serotypes are defined by particular combinations of O (lipopolysaccharide) and H (flagellar) antigens.^8^ The identification of DEC pathotypes and serotypes, which serve as critical strain markers in outbreak analyses and epidemiological surveillance, follows well-established protocols. These protocols generally involve three steps: 1) conventional culture and biochemical methods to obtain and characterize *E. coli* isolates;^8,9^ 2) molecular methods to detect virulence genes specific to five major DEC pathotypes, including *eae*, *escV* or *bfpB* for enteropathogenic *E. coli* (EPEC); *stx1* or *stx2* for Shiga toxin – producing *E. coli* (STEC); *lt*, *stp* or *sth* for enterotoxigenic *E. coli* (ETEC); *aggR*, *pic* or *astA* for enteroaggregative *E. coli* (EAEC); and *ipaH* for enteroinvasive *E. coli* (EIEC) and *Shigella*,^10–12^ and 3) serotyping using an antiserum panel against both O and H antigens.^7,13,14^ Notably, *Shigella* species can be regarded as members of the EIEC pathotype based on their virulence attributes.^15^ However, the manipulations involved in each of these three steps are lengthy and labor-intensive. Furthermore, most laboratories worldwide possess only a subset of the necessary antisera, which cover over 180 O antigens (O1 to O187) and 53 H antigens (H1 to H53).^16^ Consequently, 47.5% to 82.5% of *E. coli* isolates obtained from diarrheal patients cannot be practically serotyped.^16–18^

The association between serotype and pathogenicity of *E. coli* has been extensively explored. As early as 1949, EPEC was epidemiologically shown to be the etiological agent in frequent and very severe outbreaks of infantile diarrhea.^19^ Initially, relatively few O groups (O111 and O55) were found to be associated with diarrhea outbreaks, and several other serotypes were later on added to the list.^20^ A notorious example is the serotype O157:H7, which produces cytotoxic enterotoxins (Shiga toxin, stx) that target endothelial cells of vessels and are responsible for hemolytic uremic syndrome (HUS).^21^ Other stx producing *E. coli* serotypes also exist, such as O26, O45, O103, O104, O111, O121, and O145.^22,23^ Owing to its high immunogenicity, the O-antigen of *E. coli* is considered a promising vaccine target,^24–28^ So far, however, only a few *E. coli* serotypes have been consistently associated with diseases,^14,17^ and the correlation between serotype and pathotype is not always reproducible.^29,30^ Whether an inherent association exists between DEC serotype and pathotype remains an open question that urgently needs to be addressed.

To explore the association of *E. coli* O and H serotypes with pathogenicity, we developed a highly multiplexed, real-time PCR scheme, termed the MeltArray *E. coli* serotyping (*EST*) assay. This assay can simultaneously distinguish the five major DEC pathotypes and all available O and H groups in a single test. The utility of the MeltArray *EST* assay was demonstrated through direct analysis of anal swab samples in both spike-in validation experiments and a retrospective study. We then applied the MeltArray *EST* to investigate the association between pathotype and serotype of *E. coli* in a multi-province study and further to differentiate the four major species of *Shigella* from EIEC.

## 2. Materials and methods

### 2.1. Study design

The objective of this study was to explore a clinically applicable approach to identify all *E. coli* serotypes and demonstrate the association between serotypes and DEC pathotypes. We developed a molecular serotyping assay with the capacity of identifying all known antigen-encoding genes of O and H serotypes of *E. coli* by using 229 plasmids containing the insertion sequences of each target (Table S1) and 176 reference strains collected by the Shenzhen Centre for Disease Control and Prevention (CDC) (Table S2). Simulated samples were used to examine the ability of the assay to identify serotypes directly from fecal samples, followed by a retrospective study with a focus on the utility of the assay for culture-independent outbreak detection. After that, we used the assay to explore the association between serotypes and five major DEC pathotypes by testing 637 isolates in a multi-province study (Table S3), and went further to confirm this relationship by performing whole genome sequencing (WGS) analysis of 166 DEC isolates from EnteroBase (Table S4). Furthermore, we examined the association between *E. coli* serotypes and four major species of *Shigella* by testing 257 *Shigella* isolates (Table S5) and 18 EIEC isolates (Table S3) in our study and performing WGS analysis of 775 *Shigella* isolates (Table S6) and 360 EIEC isolates (Table S7) from EnteroBase. In all experiments, sample sizes were determined according to previous publications and experimental experience, and investigators were blinded from the nature of strains to run the assay. Data of 93 isolates in the multi-province study were classified as nonpathogenic *E. coli* and therefore excluded from analysis. Sample sizes, experimental replicates, and statistics are given in the corresponding figures, figure legends, and data files.

### 2.2. Bacterial isolates

*E. coli* reference strains used in this study (*n* = 176, Table S2) were collected by the Shenzhen CDC. All strains were grown aerobically at 37°C by shaking at 220 rpm in Luria – Bertani (LB) broth. The O-antigenic serogroups of these strains were determined using commercial *E. coli* antisera^31^ (Denka Seiken Co. Ltd., Tokyo, Japan). Strains that did not react with any of the O antisera were classified as O-nontypeable (ONT). For comparison, each isolate was analyzed via WGS by BGI Genomics Co. Ltd. (Shenzhen, China) to examine the presence of corresponding virulence genes, O-antigen biosynthesis genes, H-antigen-encoding genes, and the *E. coli*-specific gene *yccT*.

*E. coli* strains isolated from stool samples of outpatients with diarrhea and used retrospectively for the multi-province study (*n* = 637, Table S3) were collected from five provinces of China, namely, Guangdong (*n* = 355), Henan (*n* = 106), Sichuan (*n* = 75), Jiangsu (*n* = 65), and Zhejiang (*n* = 36).

The serovars of *Shigella* isolates (*n* = 257, Table S5) were determined using commercial *Shigella* antisera (Denka Seiken),^32^ and the collection was provided by the Shenzhen CDC.

### 2.3. Plasmid construction

Target genes (detailed in Figure S1 legend) of the MeltArray *EST* assay include 12 virulence genes targeting 5 major diarrheagenic pathotypes,^11^ 163 O-antigen biosynthesis genes targeting 182 O groups,^33,34^ 53 H-antigen- encoding genes targeting 53 H types^35,36^ and one *E. coli*-specific gene, *yccT*.^37^ A total of 229 plasmids that contain target sequences of the above genes (Table S1) were synthesized by Sangon Biotech (Shanghai, China). Each insertion sequence was cloned into a pUC57 vector, and the resulting plasmids were individually transformed into *E. coli* TOP10 strain.

### 2.4. Preparation of simulated samples

Stool sample negative for the EHEC virulence genes (*stx1*, *stx2*, *escV*, *eae*) was divided into 5 equal parts weighing 200 mg each for 5 multiplex PCR amplification reactions. Serially diluted (10^5^, 10^4^, 10^3^, 10^2^, 10^1^ copies/μL, determined by digital PCR via the quantification of *yccT*) cultures (50 μL) of a O157 strain (GD-325, Table S3) or a O111 strain (GD-293) were spiked into the above prepared stool samples, and used as simulated stool samples.

### 2.5. Outbreak cases in a retrospective study

A foodborne disease outbreak occurred at a high school in Shenzhen in May 2019 and had 8 suspected patients who presented diarrhea (>3 times/day) and at least vomiting (>2 times/day) or abdominal pain. Anal swabs from 7 of them were collected by the Yantian District CDC (Shenzhen, China), and *E. coli* were isolated from 5 samples and preliminary identified as EPEC strain O44 using specific antisera.

### 2.6. DNA extraction

*E. coli* isolates, *Shigella* isolates, anal swab samples, and simulated samples were stored in a − 80°C freezer before DNA extraction. Genomic DNA of bacterial isolates was extracted using the QIAsymphony DSP DNA Mini Kit (Qiagen, Hilden, Germany) following the manufacturer’s instructions; the total DNA of simulated samples and anal swab samples was extracted using a QIAamp Fast DNA Stool Mini Kit (Qiagen). The extracted nucleic acids were stored at − 80°C before use.

### 2.7. MeltArray *EST* assay

MeltArray *EST* assay reactions were performed in a 25-μL volume, containing DNA template (5 μL), and PCR master buffer [(10 mM Tris – HCl (pH 8.0) and 50 mM KCl), 7 mM MgCl_2_, 0.2 mM deoxynucleoside triphosphates (dNTPs), 2.5 U of Taq 01 DNA polymerase (Zeesan Biotech, Xiamen, China)]. Each reaction also contained a universal primer tag, tag sequence-tailed target-specific primers, mediator probes, and universal molecular beacon reporters. Sequences and concentrations of the primers and probes are listed in Table S8. PCR and melting curve analyses were performed in a SLAN 96S real-time PCR detection system (Hongshi Medical Technology Co. Ltd., Shanghai, China) following the program below: denaturation at 95°C for 5 min; 40 cycles of 95°C for 20 s and 60°C for 1 min; 35°C for 40 min, 95°C for 2 min, 45°C for 2 min, and a final temperature increase from 45°C to 95°C at 0.04°C/step for melting curve analysis. Fluorescence intensity was measured in six detection channels [Atto 425 (450 nm), FAM (510 nm), HEX (565 nm), ROX (620 nm), Cy5 (665 nm), and Quasar 705 (705 nm)] in each step of continuous temperature increase during the melting curve analysis. Data were analyzed using the SLAN 96S real-time PCR detection system software, version 8.2.2.

### 2.8. Digital PCR

Singleplex digital PCR was performed for quantification of *E. coli* isolates in the spike-in validation experiment, and triplex digital PCR was performed for quantification of linked genes in the retrospective study. Primer and probe sequences and concentrations used are listed in Table S9. Digital PCR was performed on a TD-1 Droplet Digital PCR system (TargetingOne, Beijing, China) following the manufacturer’s instructions.

### 2.9. Whole genome sequencing

The procedures of WGS and comparative genomics analysis were described previously.^38^ Specifically, DNA libraries with an insert size of 300 bp were sequenced using either a single-end 100 bp mode (SE100) or a pair-end 100 bp mode (PE100) on a BGISEQ-500 sequencer (BGI Inc., Shenzhen, China). Raw reads were quality trimmed using SOAPnuke,^39^ assembled (depth = 0, minlen = 50) into contigs by Shovill (https://github.com/tseemann/shovill), and annotated using prokka 1.13^40^ with Swiss-Prot database of Uniprot^41^ to locate the open reading frames (ORFs).

### 2.10. Genome sequences downloaded from EnteroBase

A collection of 166 DEC genomes of common serotypes defined in our multi-province study was listed in Table S4. The genomes of 775 *Shigella* (52 serotypes, comprising four *Shigella* species, Table S6) and 360 EIEC of 45 serotypes (Table S7) were available from EnteroBase (http://enterobase.warwick.ac.uk/).^42^

### 2.11. Genome sequence analysis

For genome sequences obtained by WGS or downloaded from EnteroBase, the online tool VirulenceFinder (https://cge.food.dtu.dk/services/VirulenceFinder.) was used to examine the presence of corresponding virulence genes,^43^ and SerotypeFinder (https://cge.food.dtu.dk/services/SerotypeFinder) was used to examine the presence of O-antigen biosynthesis genes and H-antigen-encoding genes.^44^

### 2.12. Statistical analysis

Data analysis was carried out in R v.4.1.2. Pearson correlation coefficient (*r*) was used to assess the linear relationship between linked genes, and a correlation coefficient value (0.80–1.00) was defined as a “very strong positive correlation”. SP ratio (the sum of isolate number of dominant pathotype of each serotype/O group/H type divided by total number of isolates) was used to define the correlation between serotypes/O groups/H types and pathotypes.

## 3. Results

### 3.1. MeltArray *E.* *coli* serotyping (*EST*) assay for *E.* *coli*

To establish a rapid and comprehensive molecular serotyping assay for *E. coli*, we used a highly multiplexed, real-time PCR scheme termed ‘MeltArray *EST* assay’. The assay is composed of 5 multiplex PCR amplification reactions whereby reaction 1 targets 12 virulence genes and the *yccT* gene of *E. coli*, reactions 2–4 target 163 O-antigen biosynthesis genes, and reaction 5 targets 53 H-antigen-encoding genes (Figure S1a). Each target in each reaction is identified based on a unique 2-dimensional (2D) label consisting of a fluorescent dye (Fn, one of the six fluorophore types in correspondence with the detection channel of the real-time PCR thermocycler) and melting temperature (*T*_*m*_).^45^ DEC pathotypes were identified based on different combinations of 12 virulence genes (Figure S1b). Serotypes were determined based on the combination of O antigens/groups and H antigens. The MeltArray *EST* assay is capable of identifying or classifying of 182 O antigens/groups and 53 H antigens. Among them, 149 have unique O-antigen biosynthesis genes and the remaining 33 share identical or very similar O-antigen biosynthesis genes,^46^ which in this study were categorized into 14 groups (Gp1 to Gp10 and Gp12 to Gp15, Figure S1c). Meanwhile, 53 H antigens all have their unique H-antigen-encoding genes.

**Figure 1.**
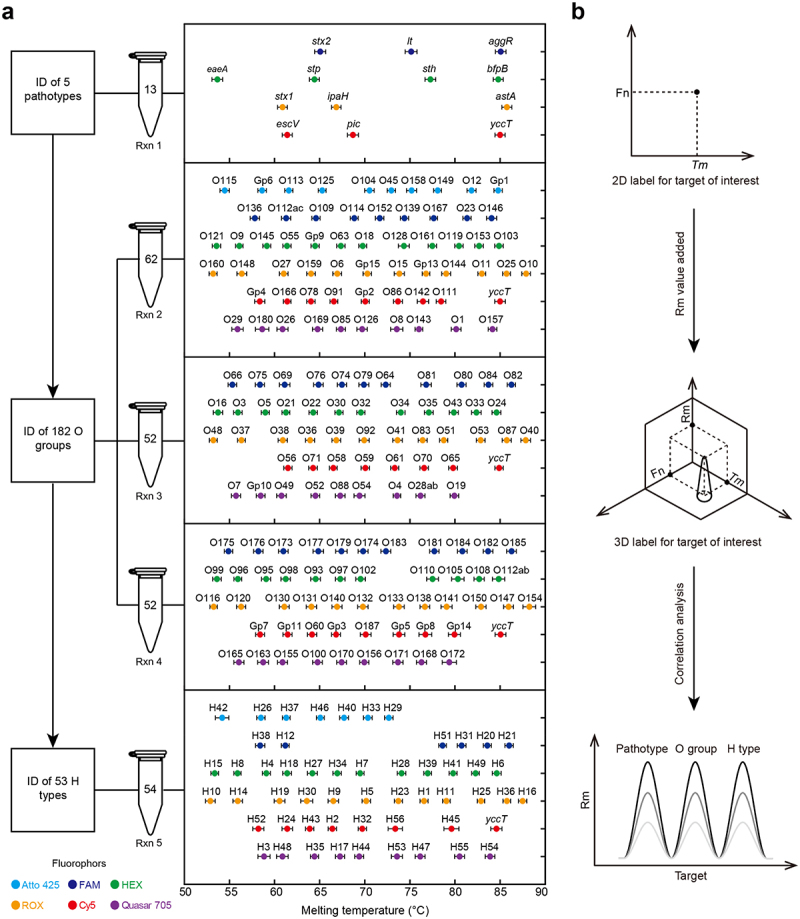
Identification (ID) of pathotypes and serotypes of *E. coli* through 5 MeltArray reactions (Rxns). (a) Setup of 5 MeltArray reactions. Rxn 1, 13-plex PCR targeting 12 virulence genes and one *E. coli*-specific gene (*yccT*); Rxn 2, 62-plex PCR targeting 61 O-antigen biosynthesis genes and *yccT*; Rxn 3, 52-plex PCR targeting 51 O-antigen biosynthesis genes and *yccT*; Rxn 4, 52-plex PCR targeting 51 O-antigen biosynthesis genes and *yccT*; Rxn 5, 54-plex PCR targeting 53 H-antigen encoding genes and *yccT*. In each reaction, the target genes are identified based on 2D labels comprising fluorophore types (Fn) and melting temperatures (*T*_*m*_). The fluorophore types are indicated by the colours of the solid circles, and the *T*_*m*_ values are shown as averaged *T*_*m*_ values ±3-fold the standard deviations (*n* = 8) using a collection of 229 plasmids with the concentration of 10^4^ copies/μL. (b) Conceptual illustration of a 3D label comprising Fn, *T*_*m*_, and melting peak height (Rm) for correlation analysis among pathotypes, O groups and H types. The 13 target genes are: *eae*, encoding intimin for *E. coli* attaching and effacing; *escV*, gene on the locus of enterocyte effacement (LEE), encoding a type III secretion factor; *bfpB*, bundle-forming pilus B; *stx1*, shiga-like toxin I; *stx2*, shiga-like toxin II; *lt*, heat-labile enterotoxin; *stp*, heat-stable enterotoxins initially discovered in the isolates from pigs; *sth*, heat-stable enterotoxins initially discovered in the isolates from human; *aggR*, aggregative adhesive fimbriae regulator; *pic*, encoding a protein involved in intestinal colonization; *astA*, enteroaggregative heat-stable enterotoxin A; *ipaH*, invasive plasmid antigen H-gene; *yccT*, a gene encoding a conserved protein in *E. coli* with an unknown function.

A series of evaluations were performed to assess the analytical performance of the MeltArray *EST* assay. Reproducibility analysis was performed using 229 plasmids containing the insertion sequences of each target. MeltArray *EST* showed a maximum coefficient of *T*_*m*_ with variations lower than 0.4%, enabling all targets to be unambiguously distinguished (Figure 1(a)). When testing 176 reference *E. coli* strains, the assay exhibited 96% concordance with traditional antisera-based serotyping and 100% concordance with whole-genome sequencing (WGS) (Figure S2). The limit of detection (LOD) was determined to be 500 copies/reaction (Figure S3). LOD analysis also showed that the concentration of each target was proportional to the height of its melting peak (Rm); Rm values were thus used to define the abundance of the target as a third dimension of the target label (Figure 1(b)), becoming 3D together with Fn and *T*_*m*_. Assuming that the copy number ratio of O-antigen biosynthesis genes, H-antigen-encoding genes, and virulence genes is fixed in *E. coli*, the combined use of Fn, *T*_*m*_, and Rm helps define the exact O:H serotype of a pathogenic *E. coli* strain. This was true because Rm values of these genes identified by 2D label were proportional to their copy number in each clinical sample, especially with disease outbreaks in which all patients were affected by the same *E. coli* strain. Thus, the MeltArray *EST* assay can be used for culture-dependent identification of DEC based on a 2D label comprising Fn and *T*_*m*_, and for culture-independent identification based on a 3D label comprising Fn, *T*_*m*_, and Rm.

### 3.2. Culture-independent identification of simulated samples

To examine the potential of the 3D label to identify serotypes directly from fecal samples, we spiked into each different normal fecal sample with either enterohemorrhagic *E. coli* (EHEC) O157:H7 or EHEC O111:H8 at a series of final concentrations (ranging from 10^5^ to 10^1^ copies/μL, quantified by digital PCR for *E. coli*) to simulate actual fecal samples from different patients containing varying numbers of *E. coli*. The MeltArray *EST* assay successfully detected the spiked-in and expected EHEC-specific (*stx1*, *stx2*, *escV*, and *eae*), O157 and H7 genes. Additionally, it detected 10 O groups and 3 H types other than O157 and H7 that preexisted in the same normal fecal sample (Figure S4a, group 1). Hence, the O157:H7 strain could not be identified by 2D label comprised of Fn and *T*_*m*_ only, owing to the presence of mixed O groups and H types in the simulated sample. This was also true of the second type of simulated samples in which the O111:H8 strain could not be identified by the 2D label, owing to the background O groups and H types when a different fecal material was spiked (Figure S4a, group 2). However, when Rm values as the 3^rd^ dimention (thus, 3D label) were additionally used for correlation analysis between the genes detected in each fecal sample, the Rm values of EHEC-specific genes (*stx1* or *stx2*, *eae*, and *escV*) were proportional to the Rm values of the serotype-specific genes of O157 and H7 in group 1, and O111 and H8 in group 2 (Figures S4b and S4c), thereby enabling specific identification of the exact serotype of EHEC spiked into the fecal sample.

### 3.3. Direct identification of the outbreak strain in a retrospective study

**Figure 2.**
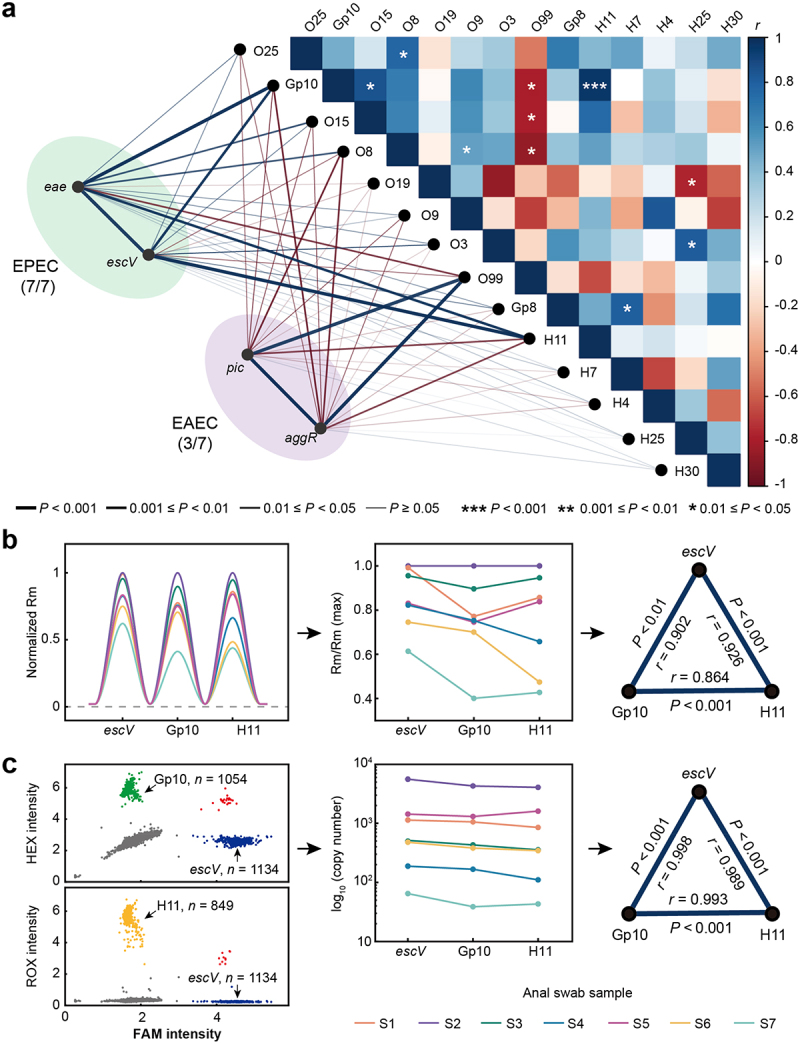
Investigation of a food poisoning outbreak in a retrospective study. (a) Correlation analysis between the melting peak height (Rm) of identified genes. Pearson correlation coefficient (*r*) was indicated by the colour of the lines or the heat map where blue indicates positive correlation and red indicates negative correlation. (b) MeltArrayArray *EST* procedure to conduct correlation analysis of the Rm values of the *escV*, Gp10, and H11 marker genes from 7 samples (S1 to S7). The three panels shown from left to right are: normalized melting peaks, normalized Rm values, and correlation analysis of the Rm values of the three marker genes. (c) Digital PCR procedure to conduct correlation analysis of the Rm values of the *escV*, Gp10, and H11 marker genes. The three panels shown from left to right are: triplex digital PCR raw data from sample 1 (S1), copy numbers of the marker genes from 7 samples (S1 to S7), and correlation analysis of the copy numbers of the maker genes.

To test the ability of MeltArray *EST* in identifying serotypes directly from clinical samples, we retrospectively analyzed stored anal swab samples collected in 2019 during a foodborne outbreak involving 7 patients from a high school in Shenzhen. EPEC-specific genes (*escV* or *eae*) were detected in all 7 samples (Figure S5). Upon examining the Rm values of all melting peaks, we observed a strong positive correlation between the Rm values of these two genes and the serotype-specific genes Gp10 and H11 (Figure 2(a)). When comparing the melting peaks of *escV*, Gp10, and H11 in the 7 samples, their normalized Rm values were found to proportionally increase or decrease across different samples, showing direct correlations among the three genes (Figure 2(b)). These correlations were further confirmed by digital PCR, which demonstrated that the gene copy numbers of *escV*, Gp10, and H11 were nearly equal in each sample (Figure 2(c)). Additionally, strong positive correlations (*r* ≥ 0.989) were observed among the copy numbers of these three genes (*escV*, Gp10, and H11), indicating that their coexistence in the genome of the outbreak strain. We thus conclude that the outbreak strain is EPEC Gp10:H11.

Isolates cultured from patient samples and subsequently subjected to the MeltArray *EST* assay yielded results consistent with those obtained directly from the anal swab samples without culturing. Sanger sequencing of the isolated strains using primer pairs for Gp10 and H11^34, 36^ further confirmed the presence of both Gp10- and H11-specific sequences, validating the results of our assay performed directly on the anal swab samples. Notably, the entire investigation process using the MeltArray *EST* assay, from the initial anal swab samples to the final results, was completed within 4.0 hrs (1.0 hr for nucleic acid extraction and 3.0 hrs for the assay), significantly shorter than the culture-dependent serotyping procedure, which typically takes at least 5 days.

To further corroborate our results with traditional serotyping, we retrieved the outbreak investigation records and found that the causative strain was initially identified as EPEC O44 by the local district CDC using an antisera-based serotyping method. However, the Shenzhen municipal CDC later corrected this to an O-nontypeable strain, as no agglutination was observed against O44 or any other available antisera. No H-type serotyping was performed at either the district or municipal CDC. This inconsistency in serotyping result by different institutions, along with the frequent lack of H serotyping in culture-based methods, highlights the advantages of molecular serotyping, which offer better reproducibility and higher resolution than conventional antisera-based method.

### 3.4. Association of serotypes with DEC pathotypes

**Figure 3.**
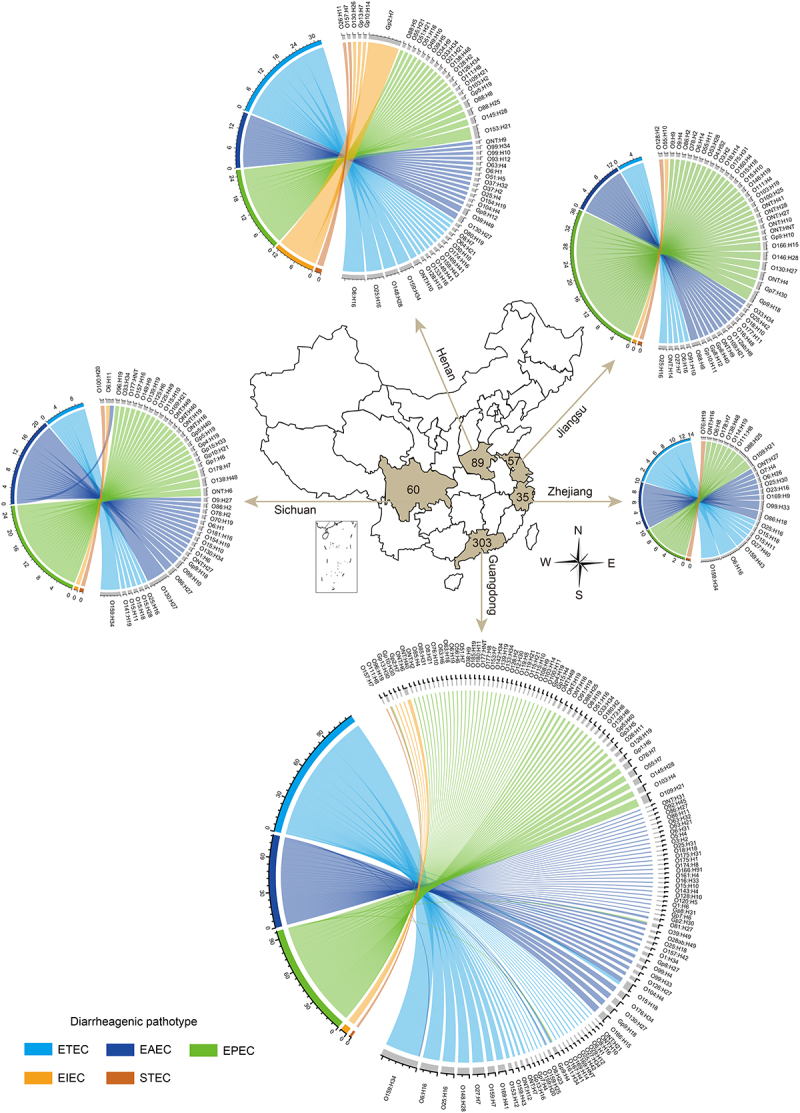
Geographical distribution patterns of diarrheagenic pathotypes and serotypes in five provinces of China.

To explore the association between serotypes and DEC pathotypes, we applied the MeltArray *EST* assay to 637 *E. coli* strains previously isolated from stool samples of diarrheal patients across five different provinces of China (Guangdong, Henan, Sichuan, Jiangsu, and Zhejiang). Of these 637 strains, 544 were classified as DEC based on the presence of virulence genes (Figure S1b). O groups and H types were identified in 93.2% (507/544) and 99.3% (540/544) of the DEC strains, respectively, resulting in an O:H serotyping rate of 92.6% (504/544). The geographical distribution patterns revealed that ETEC, EPEC, and EAEC were the dominant pathotypes, whereas EIEC and STEC were extremely rare (Figure 3). Among the 544 DEC isolates, 218 distinct O:H serotypes were identified, each comprising less than 5% of the total, except for O159:H34, which accounted for 8.3% (45/544), indicating high serotype diversity within this collection.

**Figure 4.**
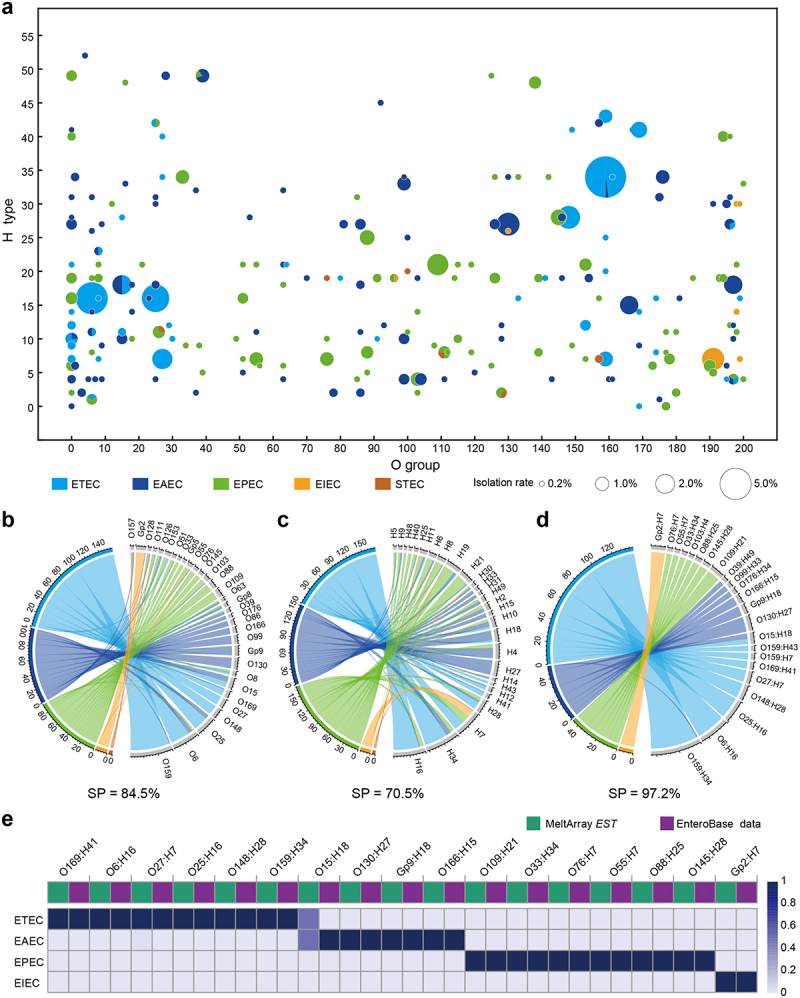
Correlation analysis of serotypes and pathotypes. (a) Distribution of serotypes and pathotypes of DEC strains in the multi-province study (*n* = 544). The numbers on the x-axis from 1 to 187 indicate O1 to O187, 190 to 194 indicate Gp1 to Gp5, 195 to 198 indicate Gp7 to Gp10, 199 indicates Gp13, 200 indicates Gp15, and 0 indicates O-nontypeable (ONT); the numbers on the y-axis from 1 to 53 indicate H1 to H53, and 0 indicates H-nontypeable (HNT). (b) Correlation of common O groups (*n* ≥ 5) with pathotypes (*n* = 375). (c) Correlation of common H types (*n* ≥ 5) with pathotypes (*n* = 522). (d) Correlation of common serotypes (*n* ≥5) with pathotypes (*n* = 250). (e) Comparison between our study results (*n* = 250) and online data analysis results (*n* = 78) with respect to the correlation of serotypes with pathotypes.

When pathotypes were superposed onto the serotypes, nearly every serotype corresponded to a single pathotype, as depicted by single-colored bubbles in Figure 4(a), indicating that these serotypes were “pure” in terms of their pathotype attribution. We introduced the concept of serotype purity (SP), defined as the percentage of isolates belonging to dominant pathotypes within each serotype. Chord diagram connecting O types/groups, H types, and serotypes (*n* ≥ 5) with the five pathotypes (Figure 4(b)−4(d)) revealed SP values of 84.5% (317/375), 70.5% (368/522), and 97.2% (243/250) for the O group, H type, and serotype, respectively. This result further demonstrates that almost every serotype exhibits a single corresponding pathotype, supporting the existence of an inherent association between serotype and DEC pathotype.

To examine whether the above conclusion is applicable to other DEC strains, we analyzed 166 whole-genome sequences of DEC strains downloaded from EnteroBase (Table S4), including the common O:H serotypes identified in in our study, EnteroBase includes a larger proportion of STEC strains compared to other pathotypes, likely due to the association of STEC strains with severe diseases. To account for this bias and the presence of the *de facto* hybrid pathotypes of STEC strains, we divided the retrieved genome sequences into two groups: a non-STEC group (*n* = 78) and an STEC group (*n* = 88). The prediction results showed that all non-STEC strains displayed fully identical associations between their serotypes and pathotypes, similar to our findings. However, one exception was noted: O15:H18 strains was classified as EAEC in EnteroBase but as either ETEC or EAEC in our collection (Figure 4(e)). The STEC strains (*n* = 88) were further subdivided into EHEC (*n* = 61) and non-EHEC (*n* = 27) strains. The EHEC group displayed a hybrid pathotype containing virulence genes from both STEC (*stx1* or *stx2*) and EPEC (*eae* and *escV*) strains, and the non-EHEC group only contained virulence genes from STEC (*stx1* or *stx2*) strains. Sequence prediction revealed that the serotype of all EHEC strains in EnteroBase was classified as EPEC in our collection (Figure S6), reflecting the hybrid nature of EHEC with EPEC. In contrast, the serotypes of the non-EHEC strains in EnteroBase were classified as both EAEC and EPEC in our collection, indicating some rare but noteworthy exceptions to the association between serotypes and pathotypes.

### 3.5. Identification of distinct serotype patterns between EIEC and *Shigella*

**Figure 5.**
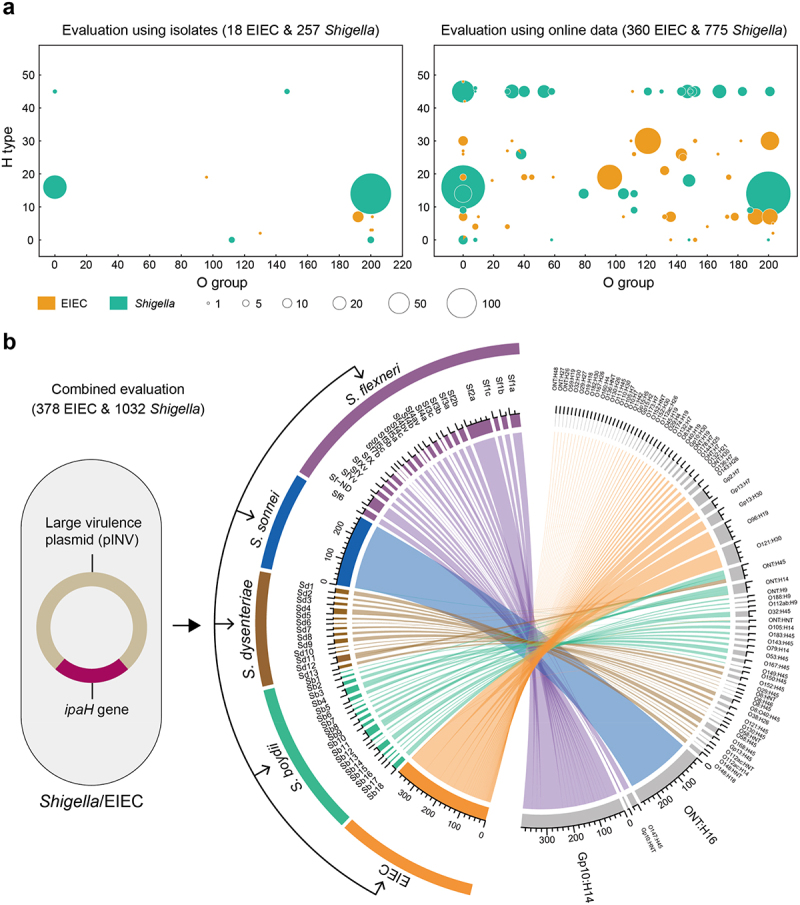
Discrepancy analysis between EIEC and *shigella* based on O:H serotypes. (a) Distribution of O:H serotypes of EIEC and *Shigella* strains. The numbers on the x-axis from 1 to 187 indicate O1 to O187, 190 to 194 indicate Gp1 to Gp5, 195 to 198 indicate Gp7 to Gp10, 199 indicates Gp13, 200 indicates Gp15, and 0 indicates O-nontypeable (ONT); the numbers on the y-axis from 1 to 53 indicate H1 to H53, and 0 indicates H-nontypeable (HNT). (b) Discrepancy of O:H serotype compositions between EIEC and four major species of *Shigella*. Sf, *Shigella flexneri*; sd, *Shigella dysenteriae*; Sb, *Shigella Boydii*; ND, *Shigella* serotype non-detected.

Given the inherent association between serotype and DEC pathotype, we hypothesized that *Shigella*, despite sharing a nearly identical pathogenic mechanism with EIEC and being genetically indistinguishable from it, might possess distinct serotypes. To test this hypothesis, we applied the MeltArray *EST* assay to 257 *Shigella* and 18 EIEC isolates from our collection. Additionally, we downloaded whole-genome sequences of 775 *Shigella* isolates, representing 52 serotypes across four *Shigella* species (Table S6) and 360 EIEC isolates, representing 45 *E. coli* serotypes (Table S7), from EnteroBase for serotype predication. The assay results revealed 100% serotype purity (275/275) for our own collection (Figure 5(a)), while the EnteroBase genomes showed a near-perfect serotype purity of 99.8% (1133/1135). These findings demonstrate that *Shigella* and EIEC can indeed be differentiated based on their distinct serotypes.

We further explored whether the four major *Shigella* species could be differentiated from each other based on serotypes. Using a chord diagram, we analyzed all 378 EIEC and 1032 *Shigella* isolates from our collection, along with the sequences downloaded from the EnteroBase. Intriguingly, the four major species of *Shigella* were clearly distinguished based on their serotypes (Figure 5(b)). Specifically, the two dominant species, *S. flexneri* and *S. sonnei*, were classified into an extremely small number of *E. coli* serotypes: 93.2% (397/426) of *S. flexneri* strains were classified as serotype Gp10:H14, and 99.6% (270/271) of *S. sonnei* strains were identified as ONT:H16. We also observed a strong association (SP = 97.9%, 138/141) between the serotypes of *E. coli* and the serotypes of *S. dysenteriae*, but a relatively weak association for *S. boydii* (SP = 75.3%, 146/194). Notably, 45.4% (88/194) of *S. boydii* strains were classified as O-nontypeable, which contributed to the reduced resolution in serotype differentiation. These results suggest that an inherent association exists between *Shigella* species and *E. coli* serotypes, further distinguishing them from EIEC.

## 4. Discussion

In this study, we established a highly multiplexed real-time PCR-based serotyping scheme for *E. coli*, MeltArray *EST*, which has demonstrated its potential to directly analyze anal swab or stool samples. In a retrospective study, we successfully performed culture-independent serotyping of outbreak strains from anal swab samples. Using this novel molecular serotyping tool, we unambiguously distinguished the five major DEC pathotypes by their unique serotype patterns. Moreover, MeltArray *EST* differentiated EIEC from *Shigella* and distinguished the four major *Shigella* species based on their distinct serotype compositions. Our findings strongly suggest an inherent association between DEC serotype and pathotype.

Traditional comprehensive *E. coli* serotyping requires over 180 antisera for O antigens and more than 50 antisera for H antigens,^33,35^ which are only available in a limited number of laboratories globally.^14^ Moreover, traditional serotyping relies on isolating bacteria in pure cultures, a slow process and often impractical for clinical or outbreak investigations,^47,^^48,49^ In addition, the presence of numerous commensal *E. coli* in the intestinal tract complicates culture-independent identification of outbreak strains.^50,51^ Furthermore, agglutination tests with specific antisera are laborious, and sometimes result in cross-reactions between different serotypes, leading to ambiguous results.^52^

In contrast, molecular serotyping methods have proven effective in accurately identifying O-antigen biosynthesis genes (*wzx*, *wzy*, *wzm* or *wzt*) and H-antigen-encoding genes (*fliC* and its homologs) of *E coli*.^33,35,53–56^ However, the number of target genes detected by conventional PCR and real-time PCR assays is limited by the resolution of band sizes in gel^33,35^ and the number of fluorometric detection channels available,^53,54^ respectively. Although microarrays^55^ and Luminex microbead-based suspension array^56^ can identify dozens of serotypes, these assays require specialized equipment, various post-PCR manipulations, and are prone to amplicon contamination. By contrast, the MeltArray *EST* assay covers 12 virulence genes, 182 O serotypes, and 53 H serotypes in just five PCR amplification reactions, which can be completed in a single step with prepared nucleic acids, taking less than 3 h, and making it a highly efficient alternative to traditional antisera-based serotyping.

The correlation between DEC serotypes and pathotypes has been a longstanding question. Although certain *E. coli* serotypes are believed to be associated with specific clinical symptoms,^7,8,20,57^ traditional serotyping often fails to accurately identify the exact pathotypes, and the correlation between serotypes and pathotypes is not always reproducible.^14,29,30^ Our multi-province study demonstrates, for the first time, a near-complete correspondence between DEC serotypes and pathotypes in China. This finding is expected to aid in disease diagnosis and outbreak detection.

The O antigen, a component of lipopolysaccharide, has been considered the most promising vaccine target for *E. coli*.^28^ For example, an O157-specific polysaccharide was developed as a vaccine (O157-rEPA; lot 011094) against STEC,^58^ and conjugate vaccine candidates were produced against two of the most prevalent O serogroups (O148 and O78) of ETEC.^59^ However, studies using traditional *E. coli* serotyping to guide vaccine target selection are limited.^60^ With its 96% concordance with traditional antisera-based serotyping and alignment with findings from previous research,^33,55,61^ molecular serotyping (both MeltArray *EST* assay and WGS analysis) can be used to guide the selection of O antigens for vaccines.

Our multi-province study also provided a dynamic profile of previously unknown or notorious serotypes that require close surveillance in China. For example, STEC can cause hemorrhagic colitis and HUS.^62^ While O157:H7 is the most well-known STEC type, the prevalence of non-O157:H7 STEC remains largely unknown due to difficulties in detection. Our study identified 7 STEC isolates, including both O157:H7 (*n* = 2) and non-O157:H7 serotypes (*n* = 5), suggesting that non-O157 STEC might even have a higher infection rate than O157:H7 in some provinces of China. EHEC strains, considered a more pathogenic subset of STEC,^8^ were also identified in our study, each belonging to one of the top 7 STEC serogroups that account for over 90% of STEC infections in the United States.^22^ Additionally, we detected the EAEC O104:H4, associated with a major outbreak of HUS in Germany in 2011,^63^ in Guangdong (*n* = 3) and Henan provinces (*n* = 1), highlighting the need for close surveillance. The burden of disease caused by atypical enteropathogenic *E. coli* (aEPEC) has been increasing annually in both industrialized and developing countries, yet the population structure of this emerging pathogen is poorly understood.^64^ Our study showed that aEPEC accounted for the majority (98.3%, 169/172) of total EPEC isolates, with a significantly higher proportion than in Africa and South Asia,^2^ suggesting that aEPEC may have replaced typical EPEC as the predominant pathotype in China.

Differentiating *Shigella* strains from EIEC is crucial for clinical diagnosis and epidemiological investigations,^65–67^ The high similarities between *Shigella* and EIEC strains make this distinction challenging.^68,69^ Our study demonstrates that MeltArray *EST* assay can unequivocally distinguish EIEC from *Shigella* based on their distinct serotype patterns. This assay has the potential to directly identify an isolate as *Shigella* or EIEC in clinical settings, filling a significant gap left by traditional serotyping methods. Interestingly, although H antigens (flagellin) are rarely expressed,^14^ H-antigen-encoding genes were identified in nearly all *Shigella* and EIEC strains (98.4%, 1387/1410) in this study, which might explain why *Shigella* species could be distinguished by molecular serotyping of *E. coli*, but not by antisera-based serotyping.

Some limitations of this study should be noted. First, 33 O serotypes were classified into 14 groups (Gp1 to Gp10 and Gp12 to Gp15) based on the presence of identical or similar O-antigen biosynthesis genes,^46^ making it challenging to distinguish O serotypes within the same group using the MeltArray *EST* assay. In such cases, WGS analysis is needed to identify specific genomic differences between strains of these O groups. Second, while we attained distinct serotype patterns of enteric pathogenic *E. coli*, the serotype patterns of commensal *E. coli* in the gut remain unknown. Future studies on commensal *E. coli* could help distinguish pathogenic from nonpathogenic strains.

In conclusion, MeltArray *EST* provides a paradigm-shifting molecular serotyping tool that confirms the relationship between DEC serotype and pathotype. It provides numerous advantages over traditional antisera-based *E. coli* serotyping methods, including substantial savings in time, labor, and costs, while maintaining high sensitivity and accuracy. Our study suggests that, when harnessed with MeltArray *EST*, *E. coli* serotype can serve as a robust disease biomarker for DEC infection and a potential target for vaccine development in addition to its indispensable role in epidemiological studies.

## Supplementary Material

Supplemental Material

## Data and material availability

All study data are included in the article and/or supporting information. The sequencing data of reference strains (Table S5) are available from the NCBI sequence database under accession number PRJNA890762.
