# Supplementary material for: Molecular serotyping of diarrheagenic Escherichia coli with a MeltArray assay reveals distinct correlation between serotype and pathotype
Source: Gut Microbes. 2024 Sep 18;16(1):2401944. doi: 10.1080/19490976.2024.2401944 (PMC11529414; doi:10.1080/19490976.2024.2401944)
Supplement: Supplemental Material [file KGMI_A_2401944_SM7198.zip › Supplemental_material_clean.docx]

**Supplemental material for**

**Molecular serotyping of diarrheagenic *Escherichia coli* with a MeltArray assay reveals distinct correlation between serotype and pathotype**

Chen Du^a,b,#^, Yiqun Liao^a,#^, Congcong Ding^a^, Jiayu Huang^a^, Shujuan Zhou^a^, Yiyan Xu^a^, Zhaohui Yang^a^, Xiaolu Shi^b^, Yinghui Li^b^, Min Jiang^b^, Le Zuo^b^, Minxu Li^b^, Shengzhe Bian^c^, Na Xiao^d^, Liqiang Li^e^, Ye Xu^a,*^, Qinghua Hu^b,*^, Qingge Li^a,*^

^a^Engineering Research Centre of Molecular Diagnostics of the Ministry of Education, State Key Laboratory of Cellular Stress Biology, State Key Laboratory of Molecular Vaccinology and Molecular Diagnostics, School of Life Sciences and School of Public Health, Xiamen University, Xiamen, China;

^b^Shenzhen Center for Disease Control and Prevention, Shenzhen, China;

^c^BGI-Shenzhen, Shenzhen, China;

^d^Yantian District Center for Disease Control and Prevention, Shenzhen, China;

^e^National Clinical Research Center for Infectious Diseases, The Third People's Hospital of Shenzhen, Southern University of Science and Technology, Guangdong Provincial Clinical Research Center for Infectious Diseases (Tuberculosis), Shenzhen Clinical Research Center for Tuberculosis, Shenzhen, China

^*^Address correspondence to Ye Xu, xuye@xmu.edu.cn; Qinghua Hu, huqinghua03@163.com; Qingge Li, qgli@xmu.edu.cn.

^#^ Chen Du and Yiqun Liao contributed equally to this article.

The authors declare no conflict of interest.

**This PDF file includes:**

Supplementary Figures 1 to 6.

**Other supporting materials for this manuscript include the following:**

Tables S1 to S9 (separate files)

**
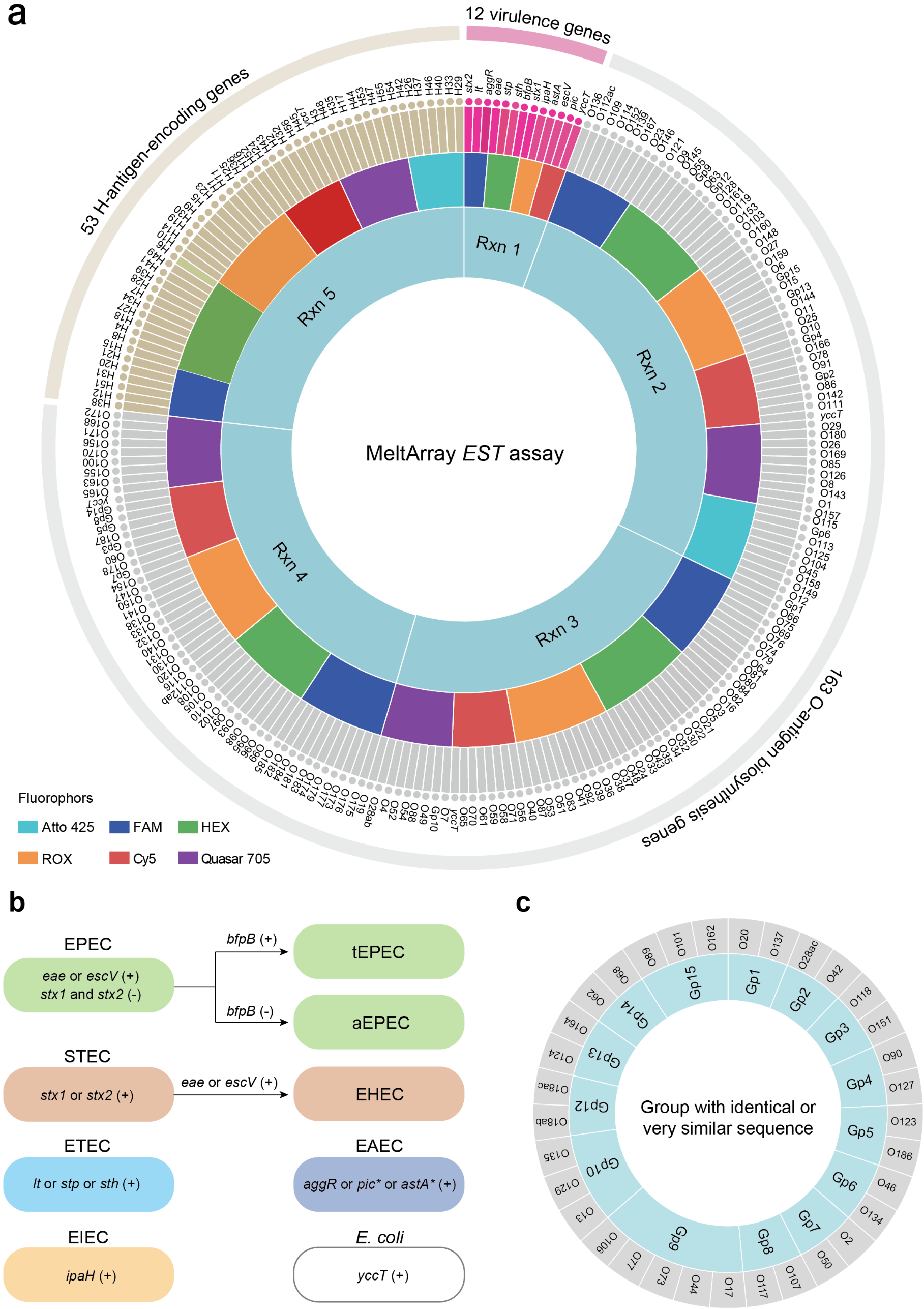
Supplementary Figure 1.** Constitution and criteria of the MeltArray *EST* system. (a) The 229 target genes included 12 virulence genes, 163 O-antigen biosynthesis genes, 53 H-antigen encoding genes, and the *E. coli*-specific gene *yccT*. (b) Criteria for the classification of five major diarrheagenic pathotypes. *When accompanied by other virulence genes, it should not be selected as the characteristic virulence gene. (c) The 33 O antigens shared identical or very similar O-antigen biosynthesis genes were placed into 14 groups (Gp1 to Gp10 and Gp12 to Gp15). Abbreviations: Rxn, reaction; *eae*, gene encoding intimin for *E. coli* attaching and effacing; *escV*, a gene on the locus of enterocyte effacement (LEE) encoding a type III secretion factor; *bfpB*, bundle-forming pilus B; *stx1,* shiga-like toxin I; *stx2*, shiga-like toxin II; *lt*, heat-labile enterotoxin; *stp*, heat-stable enterotoxins initially discovered in the isolates from pigs; *sth*, heat-stable enterotoxins initially discovered in the isolates from human; *aggR*, aggregative adhesive fimbriae regulator; *pic,* encoding a protein involved in intestinal colonization; *astA*, enteroaggregative heat-stable enterotoxin A; *ipaH*, invasive plasmid antigen H-gene; *yccT*, a conserved protein in *E. coli* with an unknown function.


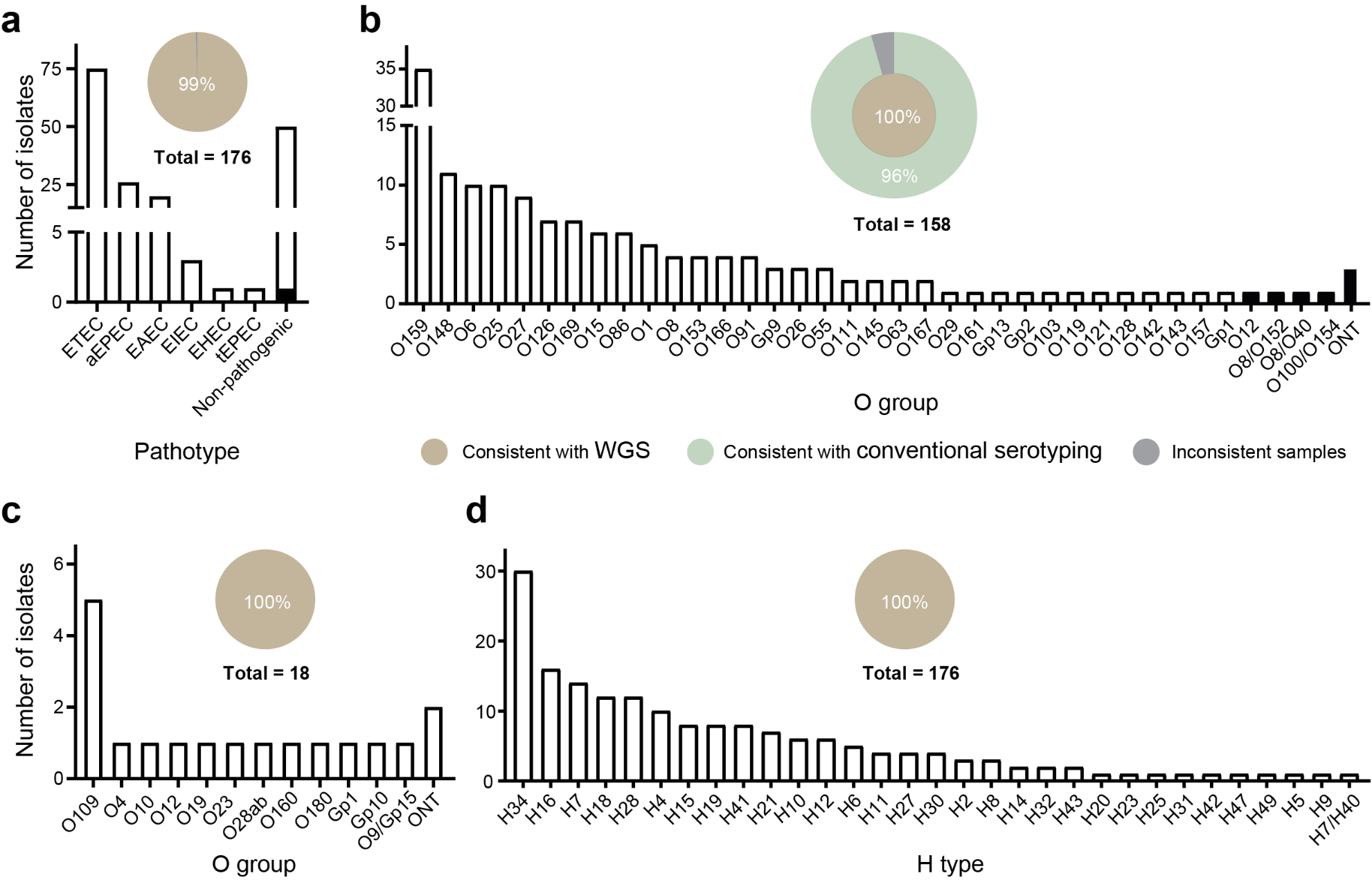
 **Supplementary Figure 2.** Distribution of pathotypes, O groups, and H types in 176 reference strains. (a) Distribution of pathotypes. Hollow bars identify isolates showing concordant results between MeltArray and whole-genome sequencing (WGS) analyses. Solid black bars identify isolates showing discordant results between MeltArray and WGS analyses. (b) Distribution of O groups in strains classified as O-typeable by conventional serotyping. Hollow bars identify isolates showing concordant results among all three methods. Solid black bars identify isolates showing discordant results between MeltArray and conventional serotyping analyses but showing concordant results between MeltArray and WGS analyses. (c) Distribution of O groups in strains classified as O-nontypeable (ONT) by conventional serotyping. Hollow bars identify isolates showing concordant results between MeltArray and WGS analyses. (d) Distribution of H types. Hollow bars identify isolates showing concordant results between MeltArray and WGS analyses.


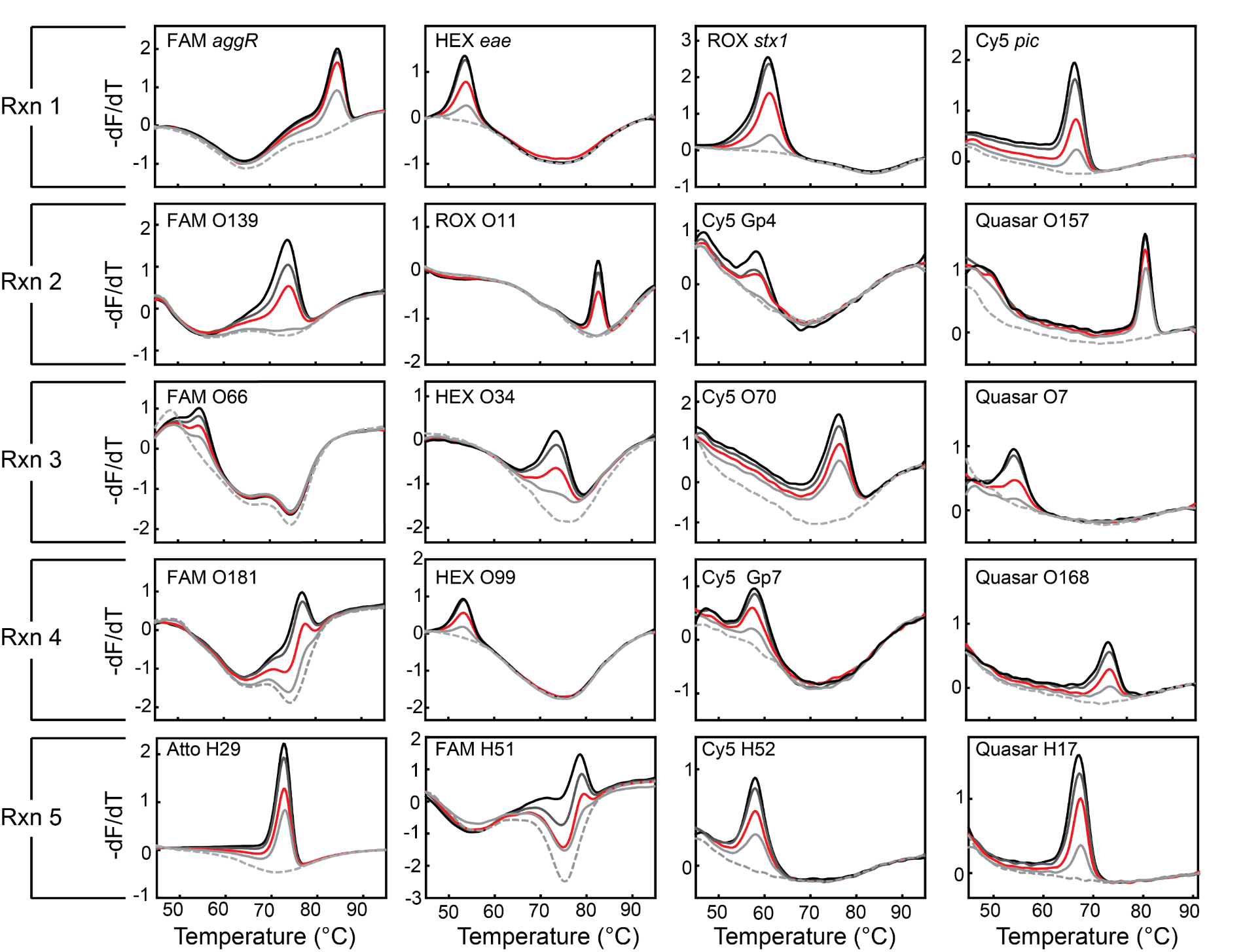


**Supplementary Figure 3.** Analytical sensitivity of 5 MeltArray reactions (Rxns). For each Rxn, melting curves of 4 representative target genes at concentrations ranging from 10^4^ to 10^1^ copies/μL are shown (grey lines) together with the melting curves representing the LOD (red lines) and no-template control (dashed lines).


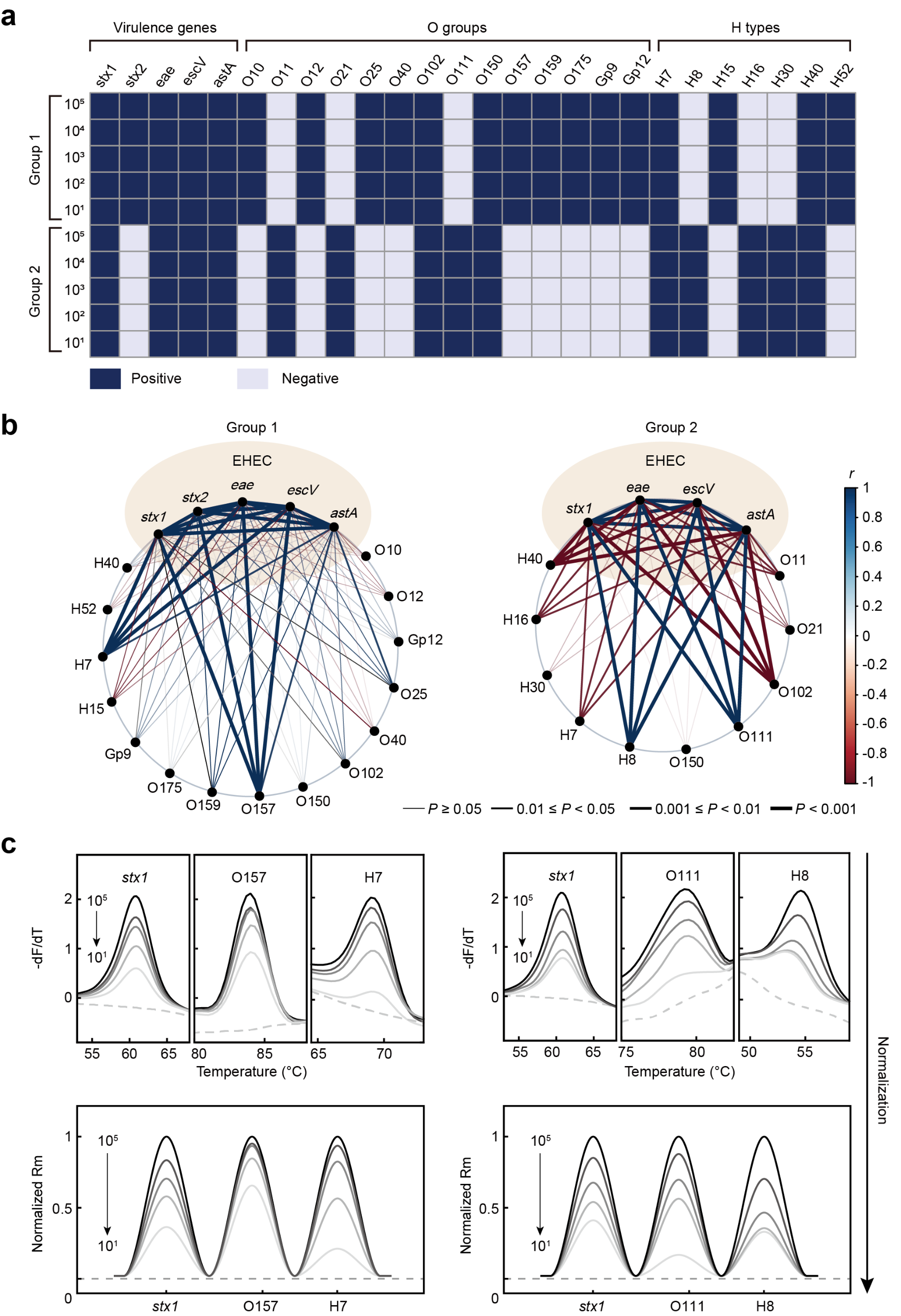
**Supplementary Figure 4.** Simulation experiments with faecal samples spiked with EHEC strains. (a) Detection results of MeltArray *EST* using 2D labelling. Two different types of samples spiked with either EHEC O157:H7 or O111:H8 strains, respectively, at final concentrations of 10⁵, 10⁴, 10³, 10², and 10¹ copies/μL. (b) Correlation analysis using the 3D labelling strategy. Pearson correlation coefficient (*r*) was used to assess the linear relationships of Rm values between the identified genes. (c) Melting peaks and normalized melting peaks of genes including *stx1*, O antigen/group, and H type of EHEC strains spiked in each type of simulated samples.


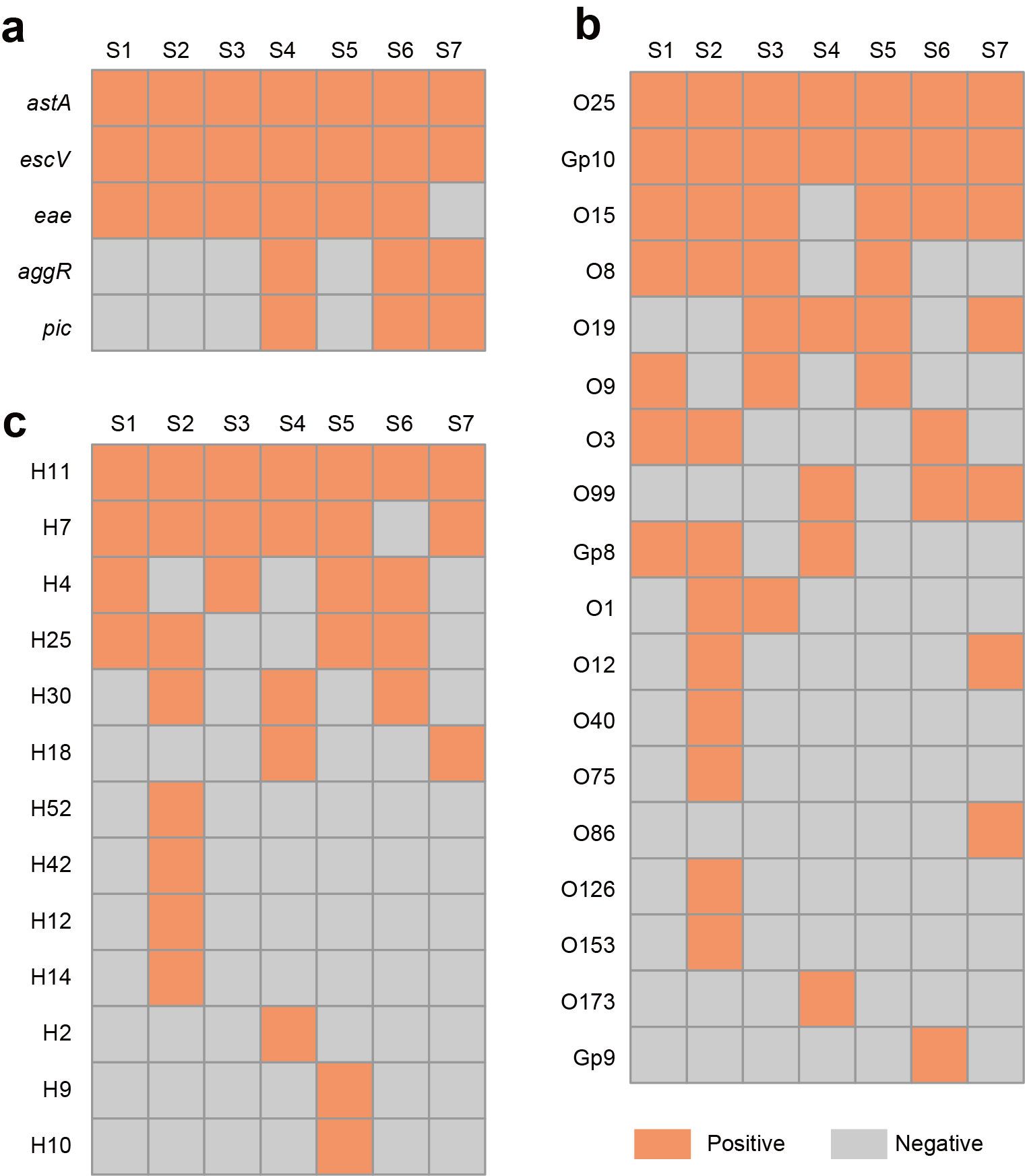


**Supplementary Figure 5.** Detection results of anal swab samples in the retrospective study. (a) Virulence genes identified in anal swab samples. (b) O groups identified in anal swab samples. (c) H types identified in anal swab samples.


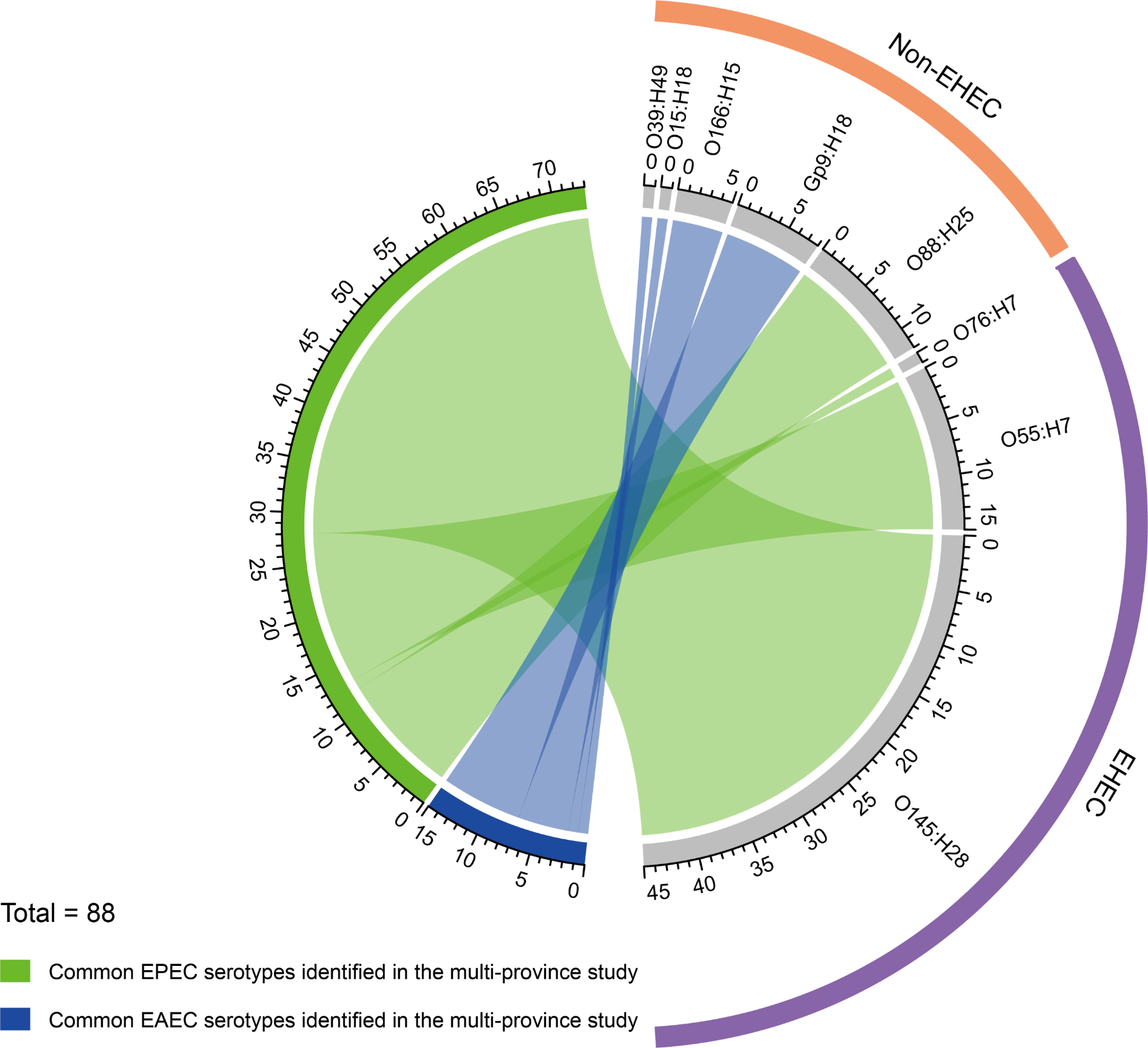


**Supplementary Figure 6.** Proportions of different serotypes distributed among the STEC group.

**Table S1 (separate file).** Insertion sequences of 229 plasmids.

**Table S2 (separate file).** Information of 176 reference isolates.

**Table S3 (separate file).** Information of 637 *E. coli* strains used in the multi-province study.

**Table S4 (separate file).** WGS data of 166 *E. coli* isolates from EnteroBase.

**Table S5 (separate file).** Information of 257 *Shigella* strains.

**Table S6 (separate file).** WGS data of 775 *Shigella* isolates from EnteroBase.

**Table S7 (separate file).** WGS data of 360 EIEC isolates from EnteroBase.

**Table S8 (separate file).** Primers and probes of MeltArray *EST*.

**Table S9 (separate file).** Primers and probes of digital PCR.
